# Quenching Thirst

**Published:** 1877-05

**Authors:** 


### QUENCHING THIRST.

Nearly a hundred years ago, Dr. Lind suggested to Captain Kennedy, that thirst might be quenched at sea, by dipping the clothing in salt water, and putting it on without wringing. Subsequently the Captain on being cast away, had an opportunity of making the experiment. With great difficulty, he succeeded in persuading a part of the men to follow his example, and they all survived ; while the four who refused and drank salt water, became delirious, and died. In addition to putting on the clothes while wet, night and morning, they may be wetted while on, two or three times during the day. Captain K. goes on to say, " after these operations, we uniformly found that the violent drought went off, and the parched tongue was cured in a few minutes after bathing and washing our clothes, while we found ourselves as much refreshed, as if we had received some actual nourishment."

The bare possibility of the truth of the statement, makes it a humanity for any paper to give it a wide publicity, since there are few readers in any hundred, who may not go to sea and be shipwrecked.

We personally know that wading in water quenches thirst, and very few readers can remember being thirsty while bathing at the sea shore, or while swimming in our rivers. When the fearful horrors of dying with thirst are remembered, and the more fearful madness which is the certain result of drinking sea water to allay thirst, it is certainly well to encourage individual experiment in this direction, and solicit an authenticated report of the same.

